# Unveiling dynamics of language visibility and vitality in café menus near Labrang Monastery

**DOI:** 10.3389/fpsyg.2024.1423672

**Published:** 2024-09-02

**Authors:** Min He, Shengnan Chen

**Affiliations:** School of Foreign Languages and Literatures, Lanzhou University, Lanzhou, China

**Keywords:** Linguistic Landscape, visibility, language vitality, translation, religion

## Abstract

**Introduction:**

This study explores the dynamics of Linguistic Landscapes (LL) in the commercial tourist district near Labrang Monastery by analyzing the use of Tibetan, Mandarin, and English on café menus. Traditionally, LL research has focused on language visibility as an indicator of vitality, overlooking contextual complexities. We challenge this approach by considering geocultural contexts, instigators’ language proficiency, and intended audience.

**Method:**

This study employed a triangulation approach, combining multiple methods of data collection and analysis. First, we conducted a preliminary observation of shop names in the region. Second, with the consent of shop owners, we comprehensively documented visual and textual artifacts both inside and outside the cafés, including signboards, menus, wall billboards, and displayed books. Third, we conducted extensive semi-structured interviews with shop owners and customers. These interviews, transcribed for analysis, provided insights into language usage preferences and decision-making processes.

**Results:**

By advocating for a holistic LL research approach, integrating qualitative insights with quantitative data, this study contributes to a deeper understanding of LL dynamics. Our findings show that geocultural contexts, instigators’ language proficiency, and intended audience all play a role in shaping language representation and vitality.

**Discussion:**

The absence of a language on signage does not necessarily signify diminished vitality but can reflect strategic decisions influenced by religious, individual and commercial factors. Beyond mere visibility, languages on café menus serve as symbolic markers of ethnic identity and reflect the functions they assume within the speech community, offering insights into language vitality across different usage contexts. This research enriches scholarly discourse on LL, particularly within the Chinese context, by emphasizing the multifaceted nature of language presence and its vitality.

## Introduction

The concept of Linguistic Landscape (LL), introduced by Landry and Bourhis (1997), provides a framework for analyzing the multilingual presence visible in public spaces. LL research typically evaluates language vitality through the visibility and prominence of languages in signage, assuming that a language’s visibility directly correlates with its vitality and status within a community (Kelly-Holmes, 2014). This approach has traditionally relied on quantitative data to measure language presence, often neglecting the complex socio-cultural and functional dimensions of language use in particular contexts.

Recent critiques highlight the limitations of solely quantitative analyses, advocating for a more nuanced understanding that considers the role of various agents and multiple modalities engaged in shaping LL in smaller territories. Researchers such as Tan (2014) and Barni and Bagna (2010) have emphasized the need to explore the genesis of LL and account for the agency of individuals within the broader socio-cultural contexts that influence language representation. Furthermore, Jaworski and Thurlow’s (2010) concept of “semiotic landscapes” calls for incorporating multimodal elements—including aural, visual, and other sensory dimensions—into LL research. Besides, research primarily focusing on cities and nations starts to narrow down to particular neighborhoods and entities like markets, food and beverage outlets, and religious institutions, urging for closer examination of the relationship between LL and people’s daily need (Pennycook and Otsuji, 2015; Theng and Lee, 2022; Coluzzi and Kitade, 2015).

To address these challenges, this study adopts a triangulation approach to investigate the Linguistic Landscapes of four cafés in the commercial tourist district near Labrang Monastery in Gannan Tibetan Autonomous Prefecture, China. By examining how these cafés use Tibetan, Mandarin, and English on their menus, the study explores the tension between “authenticity” and “commercialization” in language representation, a theme also discussed in other studies such as “authenticity and alienation” (Heller, 2010, p. 108) and “pride and profit” (Heller and Duchêne, 2007). Through interviews with café owners and a detailed analysis of signage, this research aims to shed light on how different languages are strategically deployed based on geocultural context, users’ language competence, and intended recipients, offering new insights into the interaction between language visibility and vitality.

## Linguistic Landscapes: visibility, language vitality and ethnic identity

Linguistic landscape (LL) studies have significantly contributed to our understanding of how language visibility, vitality, and ethnic identity are represented in multilingual settings. Initial research in this area has largely focused on minority language contexts to unveil power dynamics and linguistic policies within minority language contexts (Gorter, 2013). The visibility of a language is crucial as it signifies its status as a “living language” within a community and reflects prevailing language ideologies (Kelly-Holmes, 2014, p. 136). For instance, Backhaus (2006, 2007) investigated code preferences in Tokyo, revealing how Japanese in official signs reinforced existing power structures, while foreign languages on unofficial signs expressed solidarity with non-Japanese elements. Similarly, Trumper-Hecht (2010) analyzed Arabic signage in Upper Nazareth, Israel, suggesting that language display can reflect respect and recognition of minority identities within a nation. The role of LL in reflecting ethnolinguistic vitality and identity construction is also evident in Chinese studies like Nie et al. (2021), who examined the LL in Shangri-La, revealing how a recent signage policy promoted Tibetan while marginalizing other minority languages. Likewise, Nie and Yao (2022) conducted diachronic research in Lijiang Old Town, China, highlighting an increased use of the Dongba script and the tensions between the preservation of symbolic identity and the commodification of traditional language, a conflict encapsulated as “pride and profit” (Duchêne and Heller, 2012).

Recent LL studies have increasingly focused on the multimodality of language presence, exploring how various semiotic elements contribute to the construction of meaning. Scollon and Scollon (2003) introduced “geosemiotics” to explore how linguistic signs make meaning in their physical locations. Furthermore, Shohamy (2015) advocated for a multimodal approach to LL, considering images, sounds, smells, and historical contexts. Jaworski et al. (2003) and Cheung (2016) expanded LL research to include urban soundscapes, revealing how sounds contribute to the political, cultural, and social meanings embedded in different environments. In a parallel vein, Hu (2018) examined public audio announcements on Taipei’s Mass Rapid Transit System, exploring the perception and evaluation of particular soundscape by different linguistic groups in Taipei, revealing how “invisible” languages in public space find alternative presence when soundscape is considered. It was thus suggested that “all possible avenues provided by the nature of the languages” should be explored (146).

Previous studies focusing on the Chinese context have provided profound insights into the broader sociocultural contexts, where the uniquely diverse identity in this region has been subtly shaping and shaped by the language ideology embodied in LL. Song’s research on neon lights and street signs in Hong Kong examined how these signs, through their bilingual texts and semiotic images, embody social, aesthetic, and intercultural attitudes, reflecting Hong Kong’s hybrid urban culture (Song, 2020). Gu’s studies on Hong Kong’s COVID-19 related LL highlighted the critical role of multilingual communication in public health crises, revealing how translation and multilingual resources are mobilized in both physical and virtual spaces (Gu, 2022). Gu’s further research on Guangzhou Xiaobei’s LL explored the sociopolitical and policy-driven transformations in this area, illustrating the ongoing processes of de-Arabization and Sinicization while maintaining its unique identity (Gu, 2024). Zhang and Brian Chan’s works on Macau’s LL proposed a framework of separate and flexible multilingualism, analyzing how multilingual texts, such as event posters, reflect both conventional and creative language practices amidst globalization (Zhang and Chan, 2017).

This body of research highlights the necessity of adopting a multimodal approach to examining the semiotic practices via LLs in a broader sociocultural context. Existing studies have predominantly focused on the visibility and frequency of languages in signage, often overlooking how the strategic use of diverse semiotic elements—such as geocultural association, modality of communication, and target audience—reflects and negotiates complex language ideologies and commercial motives, particularly in tourist-centric ethnic-minority regions.

To address this gap, the current research integrates diverse semiotic modalities to achieve a more comprehensive understanding of their sociocultural implications. Contributing to a growing body of LL studies in China, it specifically investigates how the inclusion of English alongside Mandarin and Tibetan on signage of cafés near Labrang Monastery reflects and negotiates language visibility, vitality and ethnic identity within a tourism-driven commercial context.

## Research setting: Labrang Monastery in Gannan

As one of the ten Tibetan autonomous prefectures in China, Gannan lies on China’s northeastern border with a substantial Tibetan population of 387,134, comprising 55.9% of its local residents (Bureau of Statistics of Gannan Tibetan Autonomous Prefecture, 2021). With a rich history deeply rooted in Buddhism, Gannan is of great religious significance. Dating back to the 17th century, the Gelug Sect of Tibetan Buddhism established two monasteries, including the renowned Labrang Monastery. Over the centuries, the Gelug Sect expanded rapidly, integrating its religious doctrines into governance. Since China’s Reform and Opening-up policies in 1978, Gannan authorities, supported by national policies favoring ethnicity and religion, have endeavored to rebuild and open monasteries to meet the religious needs of local communities.

During the past four decades, Tibetan Buddhism has experienced rapid revitalization, with 181 monasteries approved for tourism by 2009, including 121 Tibetan Buddhist monasteries (Ding, 2010). Given the significant Tibetan Buddhist population, Buddhism in Gannan enjoys bountiful religious venues and clergy. It permeates public life, influencing various aspects with its doctrines, rituals, and customs, playing a unifying role among people from diverse ethnic backgrounds.

**Figure 1:**
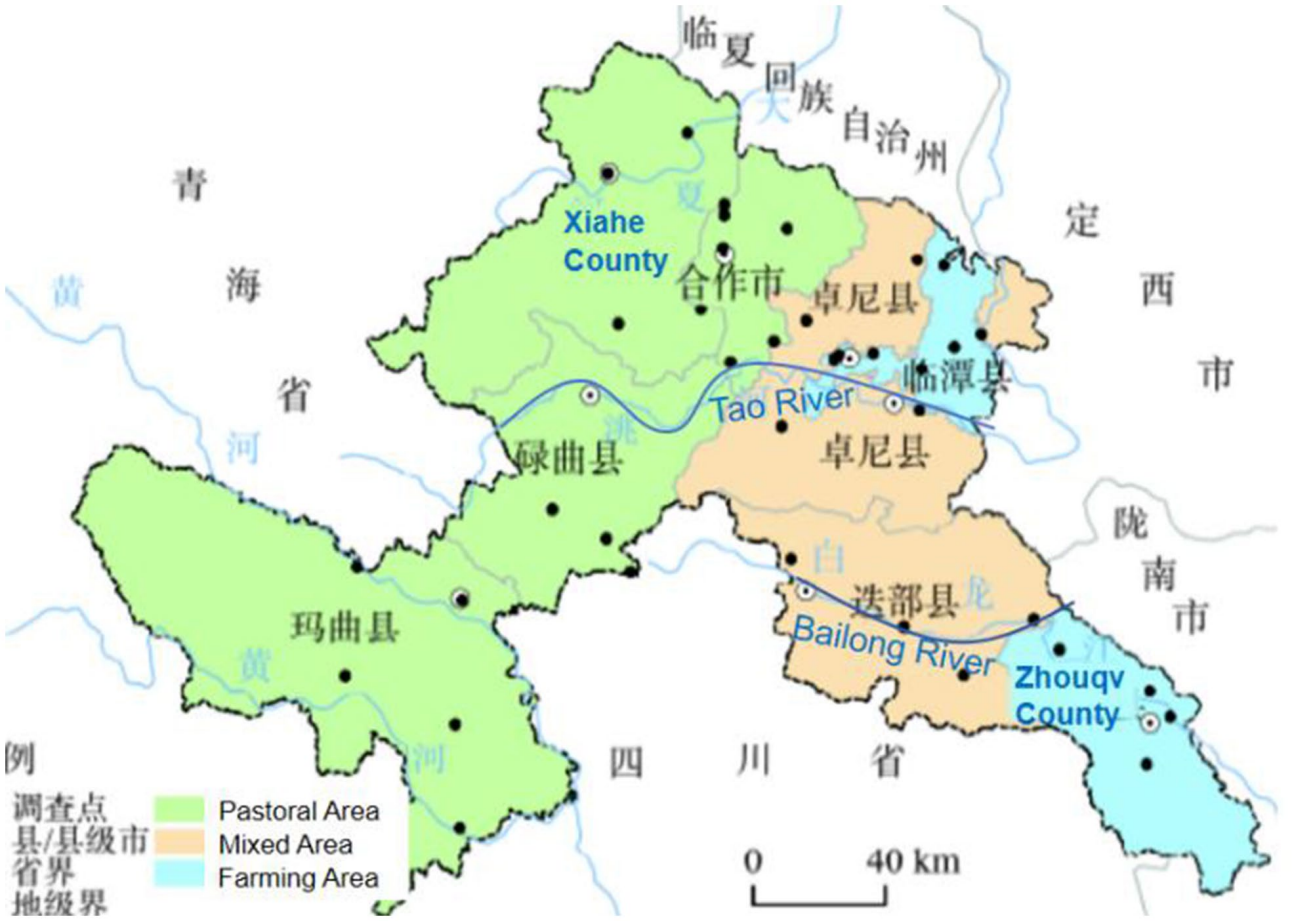
Farming and pastoral areas in Gannan, with the locations of Xiahe county and Zhouqu county.

Monasteries vary in scale, with smaller ones typically comprising fewer than 80 members, located in farming areas along the Tao and Bailong Rivers, such as Zhouqu County. In contrast, medium-to-large monasteries cluster in highland pastoral areas, like Xiahe County, home to Labrang Monastery (see Figure 1). Moreover, offerings in farming areas are typically fewer than those in pastoral areas (Ding, 2010). This situation can be explained from an ethnographic perspective: more ethnic Han Chinese migrating to farming areas due to the convenience of irrigation provided by the rivers, diluting the religious atmosphere and resulting in fewer offerings.

Labrang Monastery, preserving one of China’s best Tibetan Buddhist pedagogical systems, stands as the political and religious center of Gannan. Designated as a national key cultural relic protection site in 1982, it remains a vital venue for Buddhist gatherings and festivals, attracting millions of domestic and international tourists annually.

## Methodology

**Figure 2:**
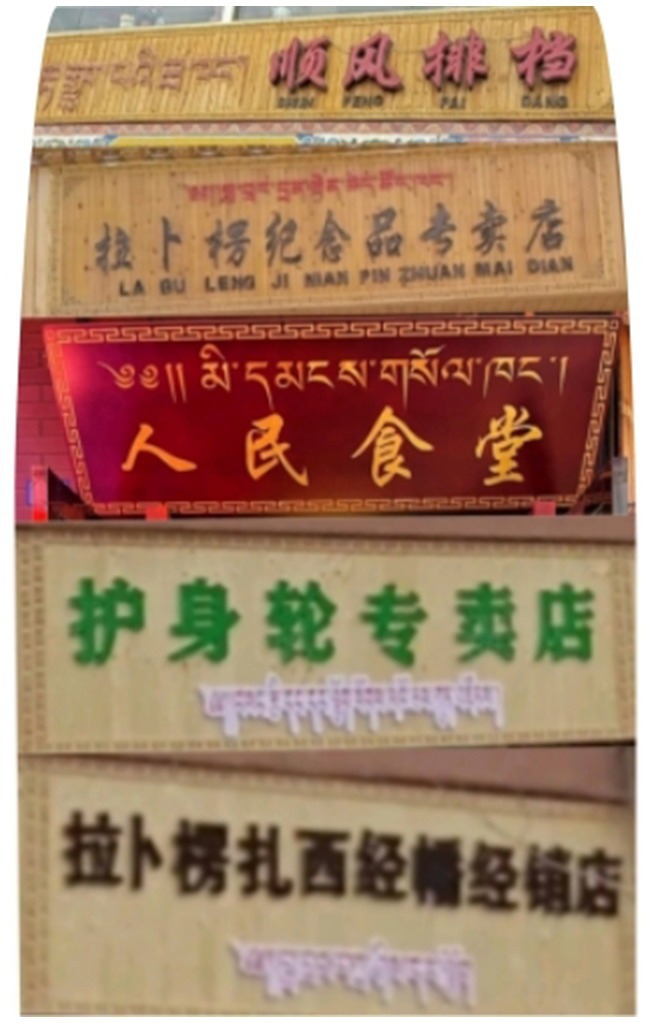
Common signboards displaying Tibetan, Mandarin, and Pinyin in Labrang tourist area.

This study investigates the linguistic landscape (LL) of four cafés near Labrang Monastery in Gannan Tibetan Autonomous Prefecture, China, and explores the underlying factors accounting for the language presence. These cafes were selected for study because they distinguish themselves by including English on their signboards in a commercial tourist district where Tibetan, Mandarin, and Pinyin (official Chinese romanization system) are typically used (see Figure 2). To enhance the robustness of our findings, we employed a triangulation approach, combining multiple methods of data collection and analysis.

**Figure 3:**
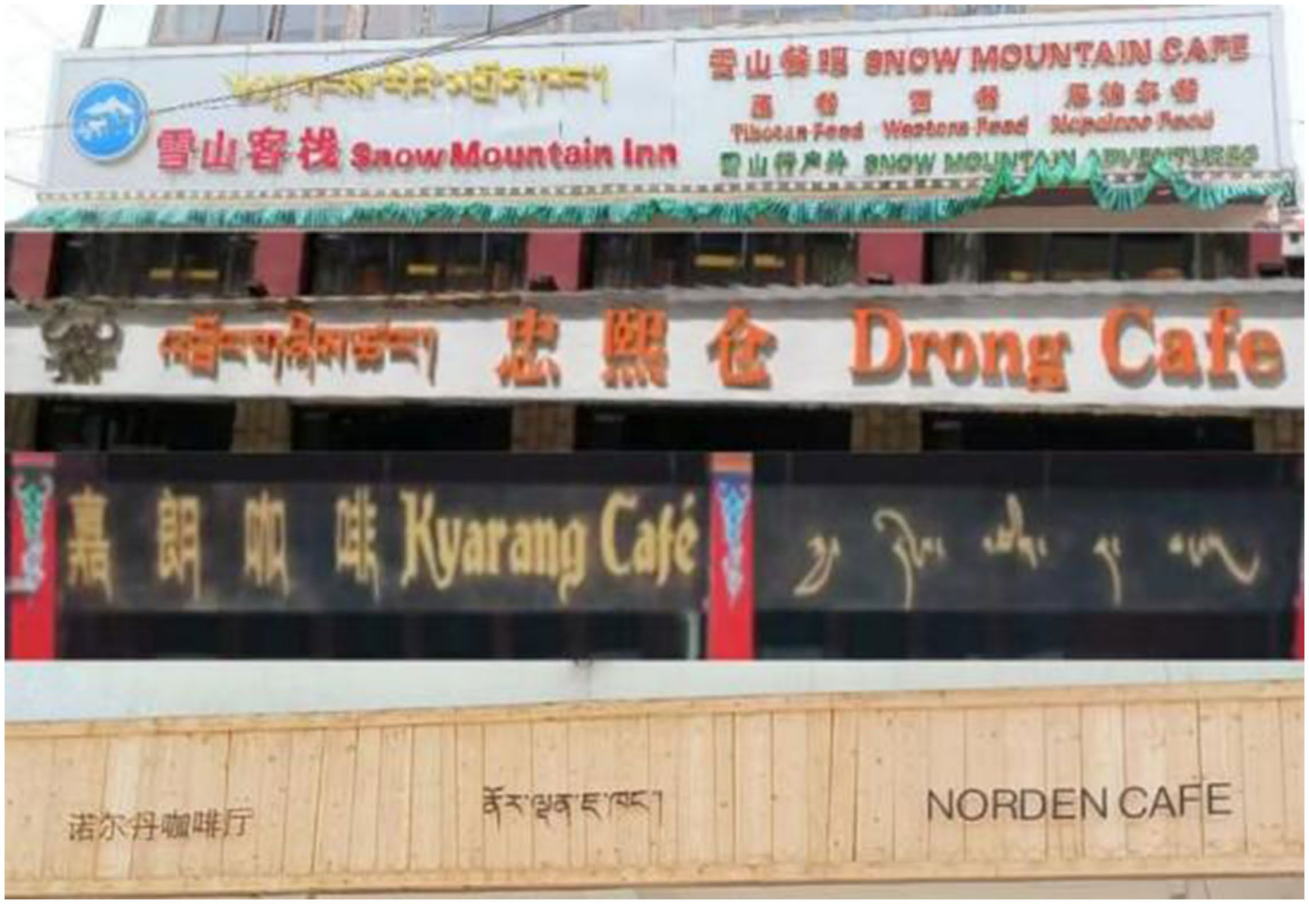
Signboards of four investigated cafés replacing Pinyin with English.

We followed three steps to collect and interpret the linguistic signage. First, we conducted a preliminary observation of shop names in the region. Most shops displayed signboards in a consistent style using only Mandarin and Tibetan and some included Pinyin, except for four cafés that uniquely incorporated English (see Figure 3). These distinctive outliers, often overlooked in quantitative analyses, prompted us to conduct a qualitative analysis to understand their divergence (Hult and Kelly-Holmes, 2019). Second, with the consent of shop owners, we comprehensively documented visual and textual artifacts both inside and outside the cafés (see Hult, 2009), including signboards, menus, wall billboards, and displayed books. This helped to capture various modes of language presence and their contextual usage. Third, we conducted extensive semi-structured interviews with shop owners and customers. These interviews, transcribed for analysis, provided insights into language usage preferences and decision-making processes. Participants’ consent was obtained, and anonymity was ensured throughout the interviews. The interview data, totaling 6 h, were transcribed into Mandarin texts using iFLYTEK Online Text to Speech Tool.

The triangulation approach—integrating observational data, visual documentation, and interviews—directed our analytical focus towards specific aspects of LL. Notably, our interview transcriptions revealed that customers predominantly engaged with signboards and menus, whereas wall billboards and book displays garnered minimal attention due to their less prominent positioning and limited functional roles in the café setting conducive to ordering. Similarly, shop owners emphasized the design and management of signboards and menus over indoor decoration. This convergence of diverse data sources facilitated a concentrated analysis of the distinctive designs found in signboards and menus.

With the triangulation method enhancing the credibility of our findings and ensuring a more nuanced understanding of the language practices in these cafés, our study aims to answer two primary questions: (1) How do shop owners display the three languages on their signboards and menus? (2) Does language visibility proportionately relate to vitality? By comparing LLs across each café, particularly the signboards and menus, we identify three dimensions of LL contributing to the representation and perceived vitality of the languages involved.

## Case study and analysis

This study delves into four cafés situated near Labrang Monastery, each uniquely incorporating English on their shop signage. The cafés in focus are Snow Mountain, Drong, Kyarang, and Norden. What distinguishes these cafés is their deviation from the norm observed in the neighbourhood, which typically features Tibetan, Mandarin and Pinyin on their signage. While such anomalies might be disregarded as outliers in quantitative studies, qualitative examination can unveil deeper narratives (Hult and Kelly-Holmes, 2019; Hult, 2009).

**Figure 4:**
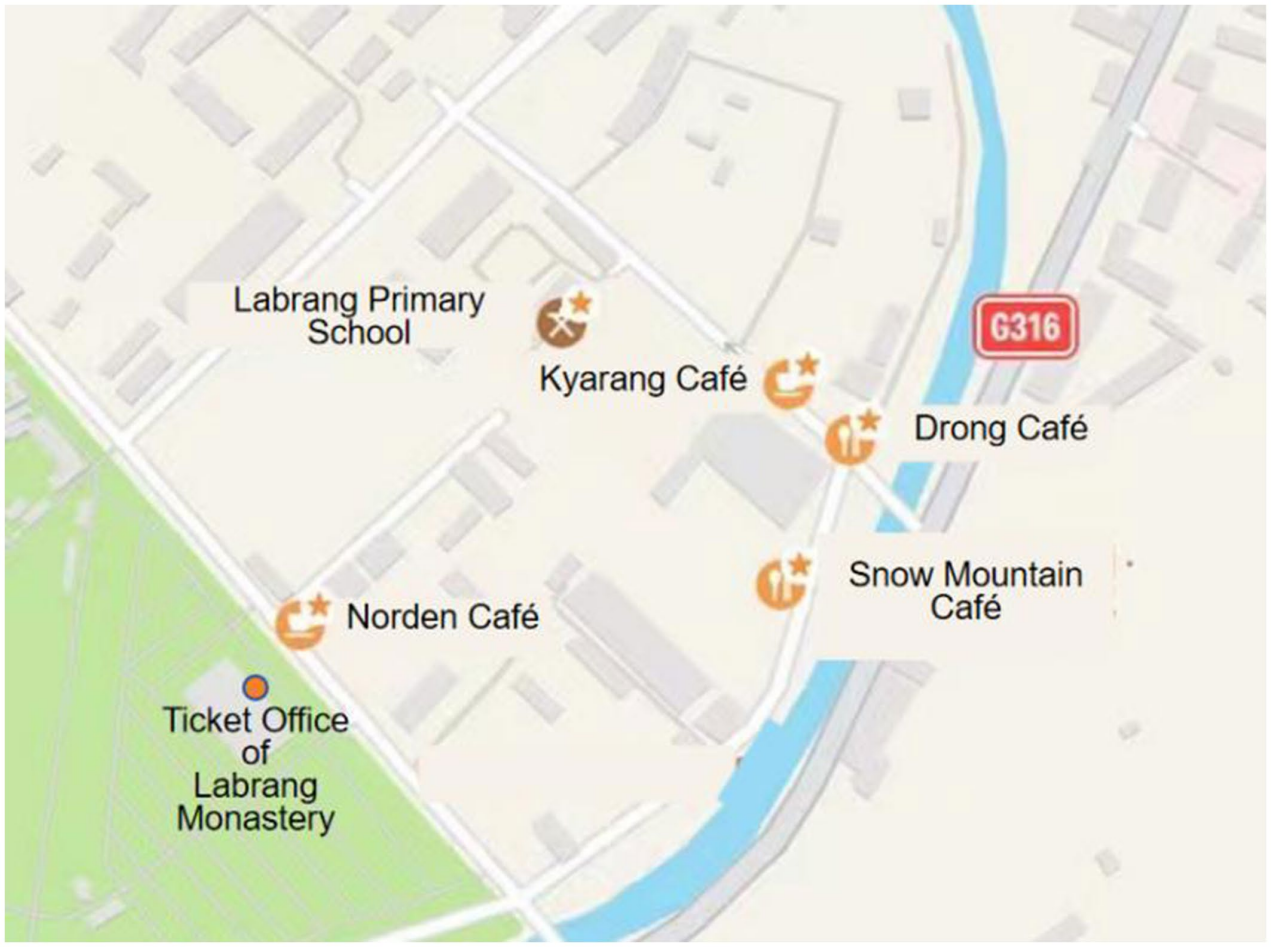
Location of four cafés and Labrang primary school around Labrang Monastery.

Norden Café stands opposite to the main entrance of Labrang Monastery, located on the bustling business street of the tourist area, whereas Snow Mountain Café, Drong Café, and Kyarang Café are situated on less-visited adjacent streets, with Drong and Kyarang being close to each other. These cafés are also in proximity to Labrang Primary School (see Figure 4). Our analysis focuses on the languages displayed on their signboards and menus and the rationale behind their language choices. Below, we analyze the Linguistic Landscapes (LLs) of the four cafés based on the accuracy, mode, and function of languages presented, and find the explanation for language behavior from interviews.

### Selective display of Tibetan: influence of Labrang Monastery’s proximity

**Figure 5:**
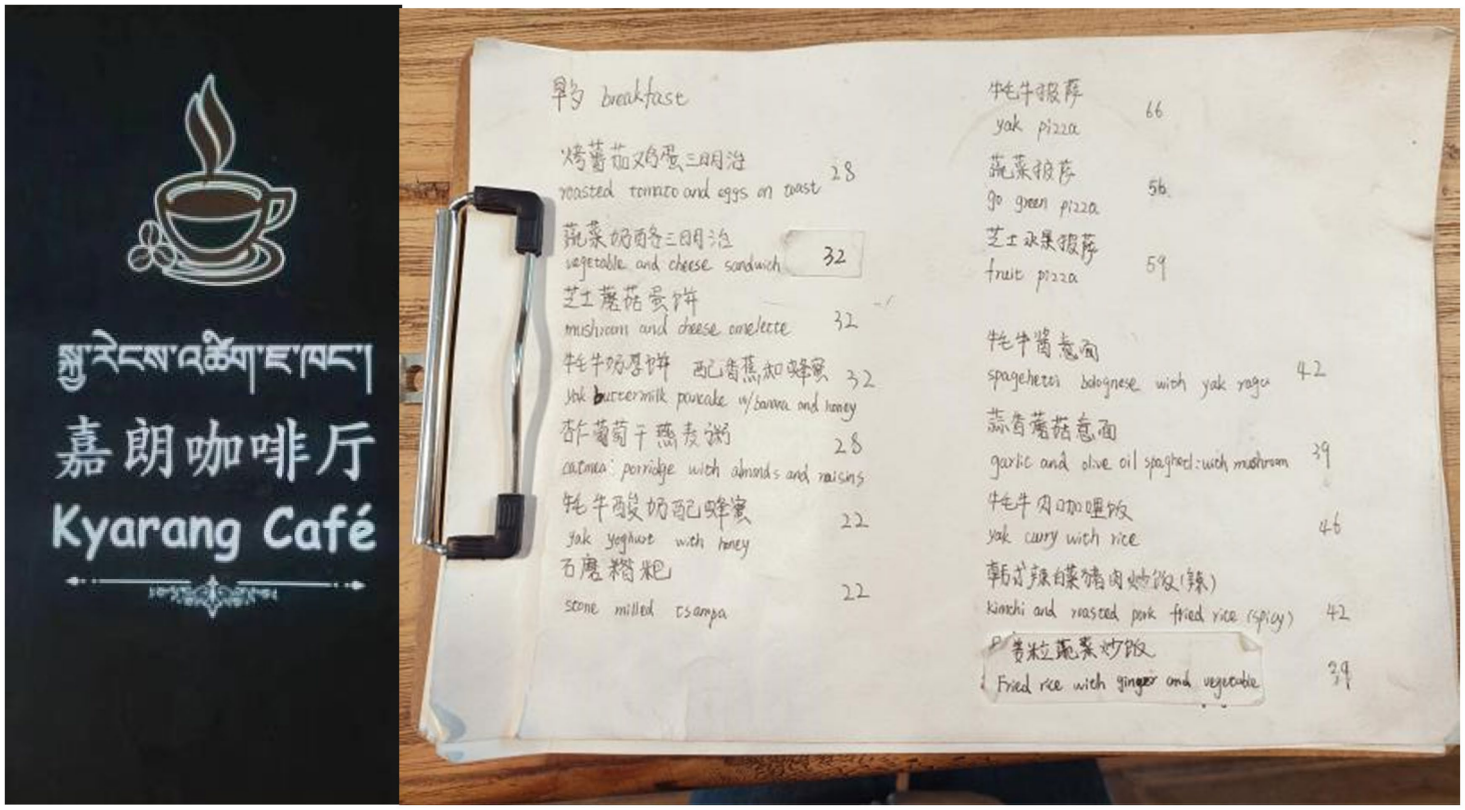
Tibetan language on menus at Kyarang Café (left) and Norden Café (right).

Since the four café in question are all located in a Tibetan area, it may be taken as a matter of course that Tibetan is displayed on their menus. But that turned out far from true. Data show that display of Tibetan is highly selective, though to varying degrees. Specifically, Snow Mountain Café features Tibetan translations for all dish names on its menu, except foreign beverages, while Drong Café offers Tibetan translations for some dishes. Kyarang Café only includes Tibetan in its shop name on the menu, whereas Norden Café notably lacks any Tibetan (see Figure 5).

A closer look at the menus and interviews with the café owners reveal that (un)translatability frequently accounts for cases where Tibetan is (not) included. When dishes are translatable, a meticulous approach was applied to Tibetan translation. For instance, the owner of Snow Mountain Café would consult Tibetan teachers from nearby Labrang Primary School and monks from the Monastery. The proximity to the School and the Monastery facilitates the process.

“We are very careful about the Tibetan translation of our dishes. After translation, we consulted Tibetan teachers from the nearby Labrang Primary School and monks in the Monastery. Of course we have no problem with daily spoken Tibetan, but when it comes to formal written Tibetan, it is better to consult those professionals*…* If you, as a Tibetan, can’t write your mother language in a correct way, the locals will look down upon you.” (Owner of Snow Mountain Café)

**Figure 6:**
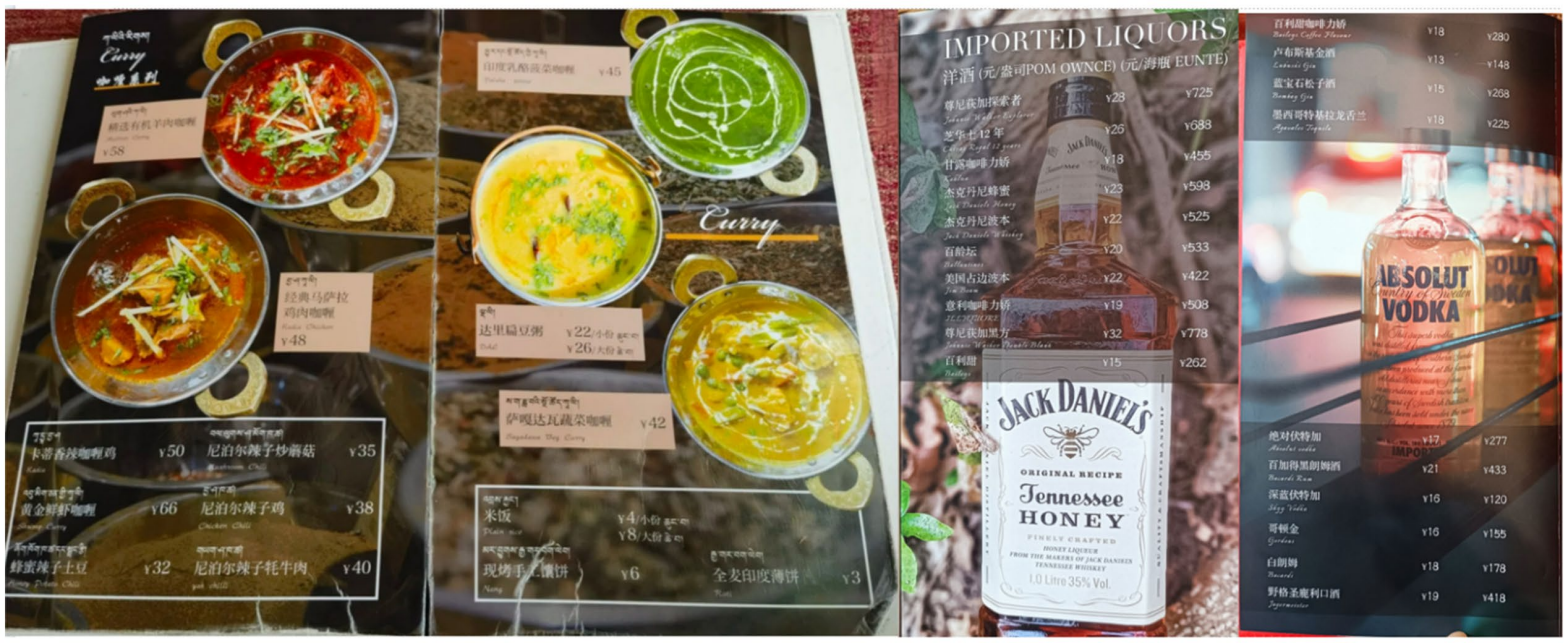
Menus at snow mountain café omitting Tibetan translations for imported liquors.

But, the translatability from English to Tibetan of foreign food and beverages varies. For instance, Snow Mountain’s speciality of “Palaka Penne” and “Honey Potato Chili” already have English names rendered in descriptive words that specify ingredients, facilitating their direct translation into Tibetan. In contrast, the names of imported liquors are typically English proper nouns, such as “Johnnie Walker Explorer” (see Figure 6). They have no equivalent in local culture, and the literal translation would lead to loss of cultural reference and provide minimal meaningful information to customers. A bad trasnlation may even do a disservice:

“In Tibetan, there are no ready translations for these Western wines, coffees, or cocktails. In daily conversation, we directly use their Mandarin names. If you cannot accurately translate them into Tibetan, it’s better not to translate them at all.” (Owner of Snow Mountain Café)

**Figure 7:**
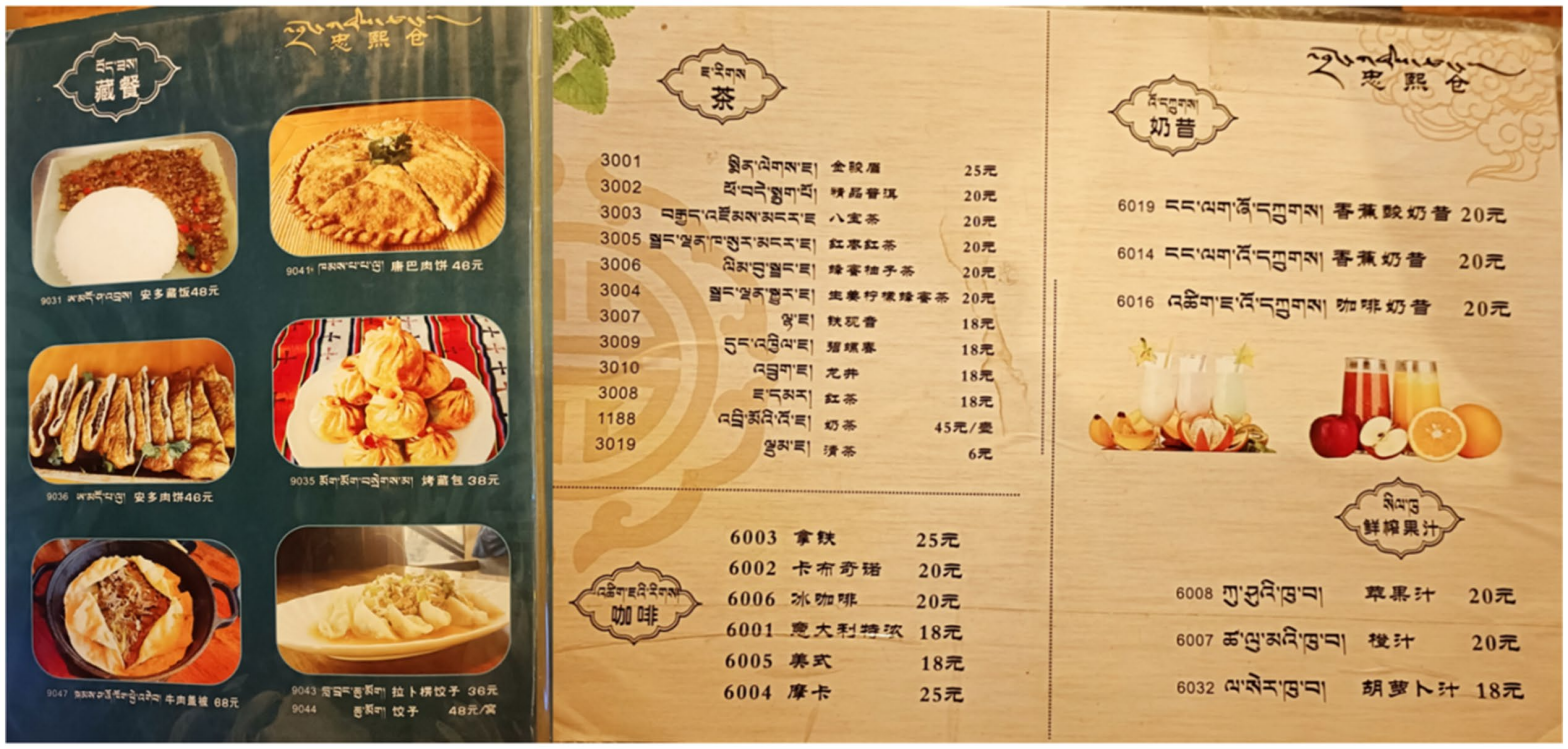
Menus at Drong Café showing Tibetan translations for traditional and foreign dishes with Tibetan equivalents, but not for foreign coffees.

Likewise, at Drong Café, the selective inclusion of Tibetan names for certain dishes also has something to do with difficulty in translation. At Drong Café, most dishes with Tibetan translations are either traditional local foods or foreign items with established Tibetan equivalents, such as the local “烤藏包” (Tibetan baked stuffed bun) and the foreign “奶昔” (milkshake). In contrast, foreign coffee varieties without Tibetan equivalents, such as “Americano,” “Latte,” and “Mocha,” are not rendered in Tibetan (see Figure 7). The owner explained:

“For those (non-local) dishes with established Tibetan equivalents, we directly use our Tibetan words. For those without Tibetan equivalents, we just omit the Tibetan translations. It is better not to translate them than to translate them incorrectly.” (Owner of Drong Café)

Like Snow Mountain Café and Drong Café, Kyarang Café’s owner explained selective inclusion of Tibetan by emphasizing the lack of interlingual equivalents in most cases. But unlike them, he also called attention to local customers’ familiarity with the menu offerings.

“Most of our customers are local friends who are already very familiar with our offerings and don’t even need to refer to the menu… Translating these Western wines into Tibetan would be meaningless because simple literal translations don’t make sense for locals. We prefer to directly describe the flavors and ingredients.” (Owner of Kyarang Café)

Norden Café, which completely omits Tibetan on the menus is, however, considering adding Tibetan to the menu. The owner noted its significance in preserving ethnic identity:

“Honestly, we are considering including Tibetan on our menu. Tibetan is our mother language, and we should take responsibility for preserving our ethnic language. And we are just across the Labrang Monastey. By displaying it on menus, we can show our ethnic culture to tourists. Besides, we can encourage our young people to learn it and enhance their awareness of their Tibetan identity.” (Owner of Norden Café)

It is observed that a significant factor motivating café owners’ shared concern for accurate Tibetan usage is their proximity to Labrang Monastery. For instance, the owner of Snow Mountain Café highlighted the religious significance of using Tibetan language accurately in the monastery’s vicinity, emphasizing its role in preserving ethnic identity.

“The monastery is very close by, which adds religious significance to the Tibetan language. For us, it represents our identity—being Tibetan.” (Owner of Snow Mountain Café)

This sentiment was echoed by the wife of the owner of Kyarang Café, who observed that the Tibetan language holds greater significance in this area compared to her hometown, where Tibetan culture is less visible and salient.

“In my hometown, Zhouqu, Han culture is more predominant than here. Although Tibetan is used on signboards there, it often appears in a smaller font compared to Mandarin. In contrast, in this area (Xiahe County), Tibetan is more prominently featured. Perhaps this is because Labrang Monastery is nearby. People here use Tibetan more frequently in their daily interactions.” (Wife of the Owner of Kyarang Café)

Zhouqu, another county in Gannan, differs from Xiahe in that it is located in a farming area rather than a pastoral one. Historically, the farming area has attracted more Han Chinese immigrants due to agriculture being their traditional livelihood. This influx has led to greater interaction between Tibetans and Han Chinese culture, which has inevitably diluted the Tibetan religious culture in the region. This situation reflects the earlier observation about the concentration of medium to large-scale monasteries and the substantial offerings in the pastoral areas of Gannan.

Therefore, it can be inferred that the selective display of Tibetan on menus relates to the high standard the café owners uphold when using Tibetan, which prevented them from accepting flawed Tibetan translations. Therefore, when cafés choose not to display Tibetan and in so doing make it somewhat less visible, it’s not because its vitality is declining, but because they are not content with *almost right* Tibetan in use if appropriate Tibetan translations are unavailable. This reflects their commitment to respecting the sacred and cultural dimensions of the language, suggesting its status as a “living language” in the religious domain (Kelly-Holmes, 2014, p. 136).

### Visibility of English: impact of spoken English proficiency in shop settings

In the cafés near Labrang Monastery, the visibility of English on menus is closely linked to the owners’ proficiency in spoken English, which determines their communication strategies. Drong Café and Kyarang Café, which previously included English dish names to accommodate their foreign clientele, chose to remove them after the COVID-19 pandemic due to the shift in customer demographics, reflecting a transition towards serving primarily domestic patrons.

“Before the pandemic, many customers were foreigners. But after that, most customers are domestic …We have employed a new chef and updated the menu, excluding English from the new menu.” (Owner of Drong Café)

“We had English dish names on menus before. Since the pandemic, our friends and domestic tourists constitute the majority of our customers. There’s no need to include English in our menus.” (Owner of Kyarang Café)

Despite this, both cafés are equipped to handle foreign customers effectively due to their owners’ English proficiency, making the visual presence of English on the menus unnecessary.

“My older brother studied abroad and speaks English fluently. He can communicate with foreign guests and recommend special dishes to them. Plus, we have pictures on the menu to help with reference.” (Brother of the Owner of Drong Café)

“My husband speaks English and has traveled internationally often. When foreign guests come, I let him take care of them. Most foreign guests usually order simple dishes directly without needing to look at the menu.” (Wife of the Owner of Kyarang Café)

In contrast, the inclusion of English on their menus at Snow Mountain Café and Norden Café is due to limited English proficiency of the owners who have to rely on others for accurate translations. At Snow Mountain Café, a Nepalese chef fluent in English provided English translations. For example, Snow Mountain Café translates “尼泊尔辣子鸡” as “Chicken Chili.” It might seem like an incorrect translation in China, where “辣子鸡” typically means “spicy fried chicken,” while “Chicken Chili” will be understood as a dish with chili being main ingredient rather than chicken. However, “Chicken Chili” is actually a popular dish in Nepal. Similarly, “辣子牦牛肉” is rendered as “Yak Chili,” and “辣子炒蘑菇” as “Mushroom Chili.” These translations, though seemingly inaccurate, reflect the authentic English usage by Snow Mountain’s staff.

Similarly, Norden Café’s English menu was prepared by the owner’s sister-in-law, who is fluent in English. The menu distinguishes between milkshakes (made from milk) and lassis (a South Asian drink made from yogurt), a distinction often overlooked in China. As the owner’s brother noted:

“Our sister-in-law is from Hong Kong and speaks English very well. However, my younger sister (the owner of Norden Café) cannot speak English. Norden Café caters specifically to tourists and needs to be more international, so our sister-in-law helped my sister translate each dish clearly into English. Foreign guests can refer to our menu without needing to ask my younger sister for details.” (Brother of the Owner of Norden Cafe)

It is found that the presence of English on café menus is directly linked to the owners’ English proficiency and their communication strategies. Cafés with owners who lack strong English skills include English translations on their menus. This is because they rely on proficient individuals to prepare these translations, helping customers understand the menu and allowing the owners to avoid direct English verbal communication. Conversely, cafés where owners are fluent in English have opted to remove English from their menus. Their ability to interact directly with foreign customers and address any menu-related queries through verbal communication eliminates the need for English on the menus as a visual aid. Therefore, the inclusion of English on these menus depends on whether the owners need this visual support due to their English proficiency, linking the presence of English directly to language competence.

### Targeted language annotations: catering to different customer bases

The language annotations on café menus provide a lens into how these establishments cater to their respective customer bases. The languages selected for providing additional details for menu items mirror the different functions of Mandarin, English and Tibetan Language perceived by the instigators.

**Figure 8:**
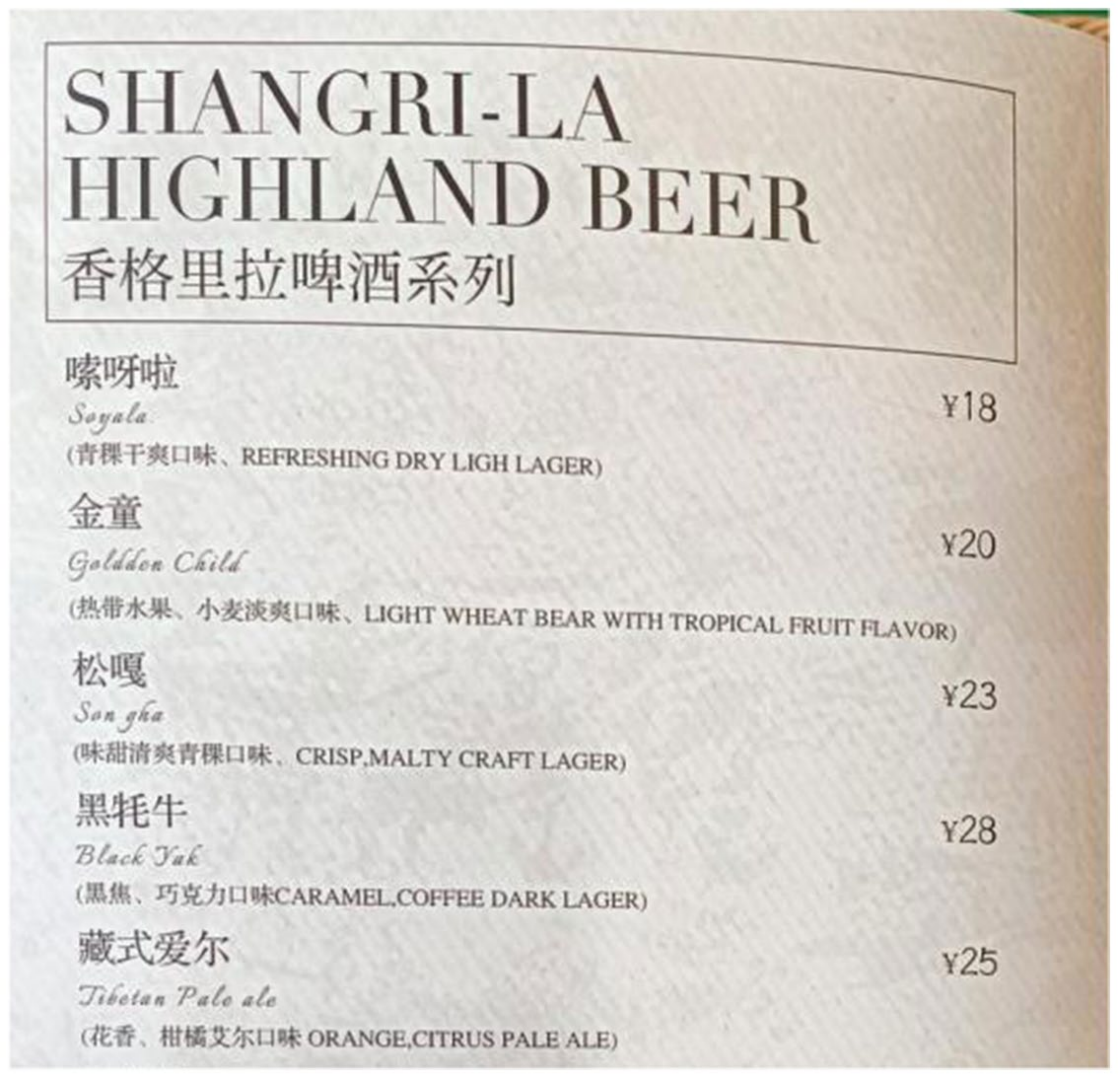
Annotations of “Shangri-La Highland Beer” series at snow mountain café: example of “Golden Child”.

**Figure 9:**
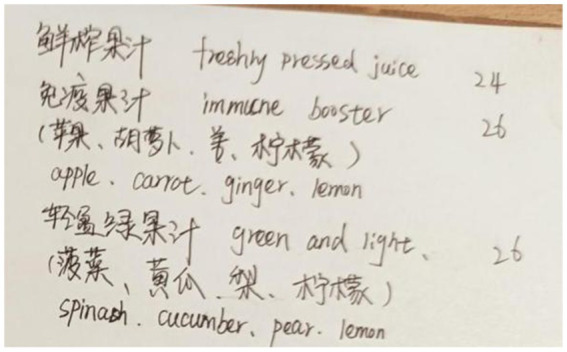
Bilingual ingredient descriptions for juice at Norden Café: example of “Immune Booster”.

Snow Mountain Café and Norden Café strategically use bilingual annotations (Mandarin and English) to cater to tourists. At Snow Mountain Café, the menu for imported beers includes detailed descriptions, like “金童/Golden Child” from the “Shangri-La Highland Beer” series, annotated as “热带水果、小麦淡爽口味/Light Wheat Beer with Tropical Fruit Flavor” (see Figure 8). Norden Café employs a similar strategy for its juice offerings, providing Mandarine and English annotations for names like “免疫果汁/Immune Booster” and “轻盈绿果汁/Green and Light.” For instance, “Immune Booster” is explained as containing “苹果、胡萝卜、姜、柠檬/Apple, Carrot, Ginger, Lemon” (see Figure 9).

The two Cafés’ decision of leaving out Tibetan in annotations is justified by their consideration that these annotated items do not target local customers who speak Tibetan.

“Local customers come here usually for our burgers and pizzas. Beers and liquours often attract tourists coming here for holiday.” (Owner of Snow Mountain Café)

“Our target customers are tourists, not locals. For staple, we only offer small portions of sandwiches and spaghetti, which obviously cannot satisfy the local appetite. That’s why we don’t include Tibetan on menus.” (Owner of Norden Café)

In contrast, Drong Café and Kyarang Café, not including any language annotations on their menus, cater mainly to local residents. According to the owners, their regular patrons are already well-acquainted with the menu items and can easily ask about any questions in Tibetan:

“Most of our patrons are local families who are well-acquainted with our home-style dishes. If they had any questions, they would ask our servers straightforward; so we don’t find it necessary to add extra explanations on menus.” (Owner of Drong Café)

“Our regular customers live nearby and are very familiar with our menu items. We use Tibetan to introduce unfamiliar items directly to them.” (Owner of Kyarang Café)

The language choices for menu annotations in these cafés reflect a strategic alignment with their intended recipients. Cafés such as Snow Mountain Café and Norden Café include Mandarin and English descriptions to cater to tourists, who primarily speak these languages, while omitting Tibetan. In contrast, Drong Café and Kyarang Café, which cater primarily to local residents, avoid additional annotations since their menu items are familiar to local customers, who prefer direct communication in Tibetan if they have any queries. This strategic use of language reflects a conscious effort to align menu presentation with the needs and preferences of their respective customer bases. Thus, the presence or absence of a language is possibly a deliberate commercial strategy based on the intended recipients.

## Discussion: language presence and vitality

In this section, we delve into the multifaceted dynamics of language presence and usage as observed in the café menus of our case study. By examining the role of geocultural contexts, language proficiency of instigators, and intended recipients, we aim to uncover how these factors influence the representation of Tibetan, Mandarin, and English in the Linguistic Landscapes (LLs) near Labrang Monastery. This discussion challenges some conventional assumptions in LL research and highlights the complexities in interpreting language presence as an indicator of language vitality.

### Geocultural association of location

The religious significance of Labrang Monastery enhances the association between accurate Tibetan usage and ethnic identity. It enhances the status of Tibetan as a living language in the religious domain and maintain its vitality in the region, despite previous national language policies favoring Mandarin. The prohibition of minority language use by national policy during the 1970s aimed to promote ethnic integration, resulting in a significant decrease in the visibility and audibility of minority languages in both public and private spheres in China (Shen and Gao, 2019). Despite a shift towards pluralism in language policy since the Reform and Opening Up period in 1978, the widespread adoption of Mandarin and the standardization of Chinese characters have posed significant challenges to the preservation of minority languages. However, the religious significance of Labrang Monastery has served as a bastion for the Tibetan language in this region, as many Buddhist sutras and mantras are exclusively recorded and taught in Tibetan. Consequently, it leads the locals to prioritize the accuracy of Tibetan language usage, sometimes to an extent that Tibetan usage is avoided when accuracy cannot be guaranteed. In this sense, the geocultural context has played a pivotal role in closely linking vernaculars with ethnic and religious identity, shaping the linguistic ideology of the local community. Contrary to common perceptions in LL research, the absence of Tibetan language on signage in this context does not indicate its poor vitality, but is justified by its religious association with ethnic identity that requires a high level of language accuracy.

This observation is further supported by previous research on the commodification of Dongba script (Nie and Yao, 2022), which also underscores the influence of religious context. The deeply ingrained beliefs within the speech community subject various stakeholders, including tourist practitioners and locals, to high stakes regarding the commercial exploitation of their ethnic language and to concerns about maintaining the “integrity and authenticity” of local religious culture (p. 23). These worries once refrained them from presenting Dongba script for a certain period of time.

This insight emphasizes the importance of considering specific geocultural contexts while gauging the language vitality through LL items. The special location of linguistic signage allows a sociocultural context where the languages involved can be uniquely associated with identity construction and negotiation, enabling a more intricate interpretation of its vitality based on its visibility. Without this contextual understanding, such critical aspects as the accuracy or authenticity of language use may be overlooked or misunderstood in LL research.

### Language proficiency of instigators

The visibility of English on menus is significantly influenced by the Café owners’ English proficiency. Specifically, the Café’s owners, who possess strong English skills, choose not to include English on their menus, relying instead on direct verbal communication with international guests. In contrast, the Cafés, where the owners have limited English proficiency, incorporate English translations on the menus to compensate for their lack of oral fluency. Their reliance on visual English representation helps them avoid the need for direct English conversation.

This phenomenon highlights a key insight for LL research that the language proficiency of LL instigators (café owners, in this case) plays a crucial role in determining the sensory mode through which a language is represented. Traditional LL research has often focused predominantly on the visual presence of languages, potentially overlooking their aural or other sensory manifestations. Nevertheless, a language’s visual absence on signage does not necessarily indicate its lack of vitality or proficiency within a community. Instead, it might reflect an effective use of alternative sensory modes, such as oral communication, where the language still thrives in practical use.

Hu (2018) supports this perspective by demonstrating that the auditory presence of languages, like Holo and Hakka in Taipei’s mass rapid transit system, can complement the visual signage and provide a more comprehensive understanding of language vitality. Thus, the visual absence of a language should not be interpreted as a sign of its diminished vitality, but rather as a possible reflection of its presence in other sensory channels.

These multimodal understandings of LL challenges the conventional notion that visual presence alone indicates language vitality and underscores the necessity of incorporating diverse sensory modes into LL research. It prompts researchers to account for instigators’ deliberate decision of particular sensory modes for LL items and consider the underlying factors like language competence. Such an approach not only enriches our assessment of Linguistic Landscapes but also aligns with the lived experiences of language users.

### Intended recipients of signage

The language annotations on café menus reveal strategic choices aligned with their target customer bases, or the intended recipients of LL items. Snow Mountain Café and Norden Café use Mandarin and English annotations to cater primarily to tourists, aligning with their business focus on non-local customers. Conversely, Drong Café and Kyarang Café, which serve a predominantly local clientele, do not include any annotations as their local customers can easily inquire about details in Tibetan. The language choices is indicative of the owners’ strategic choice based on the anticipated audience of the menus. Tourist-focused cafés prioritize Mandarin and English to facilitate interactions with visitors, highlighting these languages’ functional role in commercial contexts. In contrast, cafés serving local residents use Tibetan extensively, indicating its predominant role in daily communication among locals.

These observations suggest language presence on LL items should not be considered a sole indicator of language vitality, as the former is often well-designed to serve the intended audience in various contexts. In a tourist context, a language prevails in daily communication among locals may be absent from commercial settings targeting non-local customers. Without considering the target audience of LL, such absence of a language cannot be collected as quantitative data to measure language vitality as it subjects to the instigators’ agency prompted in a particular setting.

The role of instigators’ agency have also been discussed by Hult and Kelly-Holmes (2019) who investigated the semiotic landscape of a tailor shop uniquely using Scandinavian in the Chinatown of Singapore. The individual agency of the shop owner, situated in a specific socio-historical context, contributed to the unique display of signage, which seems an outlier in its community. Similarly, the café owners’ language choices reflect their marketing strategies and target audience, illustrating how business decisions impact language representation in commercial settings.

While traditional LL research often focuses on the frequency of a language appearing in signage, incorporating the target audience into the analysis provides a more nuanced understanding of language vitality. As Mandarin and English’s presence in tourist-oriented cafés highlights their role in a specific commercial context and Tibetan’s absence from these menus reflects its everyday use among locals, understanding language vitality requires considering various contexts, including both commercial and daily life scenarios, that target different recipients. By examining how language is used across different modalities and contexts, researchers can gain a more comprehensive view of its functional and cultural significance within a community and thus reveal its vitality.

Based on the above observations and analysis, the absence of a language on signage does not necessarily signify diminished vitality but can reflect strategic decisions influenced by religious, individual and commercial factors. By considering these complexities, this study highlights the role of geocultural contexts, language proficiency of instigators, and the intended recipients in shaping Linguistic Landscapes, enriching our interpretation of language visibility and aligning with the dynamic and context-dependent nature of language use in diverse sociocultural settings.

## Conclusion

This study has delved into the intricate dynamics shaping Linguistic Landscapes (LL) within the commercial tourist district adjacent to Labrang Monastery. By examining the use of Tibetan, Mandarin, and English on café menus, we aimed to reveal how geocultural contexts, language proficiency of instigators, and intended recipients of LL items contribute to the representation and perceived vitality of languages.

Our findings challenge traditional LL research assumptions that equate language visibility solely with vitality. We advocate for a holistic approach to LL research that integrates geocultural contexts, instigators’ agency, and audience considerations. By moving beyond quantitative approaches and embracing a multifaceted understanding of language visibility, researchers can better capture the complexities of LL dynamics between language presence and its vitality. This approach not only enriches our understanding of how languages function in public spaces, especially the ethnic-minority commercial area, but also highlights their symbolic and practical roles in shaping cultural identities and daily interactions, contributing to a growing body of LL scholarship in the Chinese context.

## Data Availability

The raw data supporting the conclusions of this article will be made available by the authors, without undue reservation.
